# Cervical cancer screening uptake: A randomized controlled trial assessing the effect of sending invitation letters to non-adherent women combined with sending their general practitioners a list of their non-adherent patients (study protocol)

**DOI:** 10.3389/fpubh.2022.1035288

**Published:** 2022-11-10

**Authors:** Delphine Teigné, Anne-Sophie Banaszuk, Charlotte Grimault, Linda Abes, Aurélie Gaultier, Cédric Rat

**Affiliations:** ^1^General Practice Department, Faculty of Médecine, Nantes, France; ^2^Research Department, University Hospital of Nantes, Nantes, France; ^3^Regional Organization in Charge of Cancer Screening Programmes, Angers, France; ^4^Research Department, Methodology and Biostatistics Platform, University Hospital of Nantes, Nantes, France; ^5^National Institute for Health and Medical Research/INSERM U1302 Team 2, CRCINA, Nantes, France

**Keywords:** screening uptake, cervical cancer screening (CCS), organized screening programmes, general practitioners, randomized controlled clinical trial (RCT)

## Abstract

**Introduction:**

Cervical cancer (CC) is the fourth most common cancer among women. It can be cured if diagnosed at an early stage and treated promptly. The World Health Organization suggests that 70% of women should be screened with a high-performance test by the age of 35. This paper reports a protocol to assess the effect of two modalities of organized CC screening programmes on CC screening uptake.

**Methods and analysis:**

*Design and setting:* The design involves a 3-arm randomized controlled study performed in a French geographic area on the west coast. A total of 1,395 general practitioners will be randomized, depending on their general practice surgeries. *Participants:* The design is based on a total of 94,393 women aged 40 to 65 years who are eligible for CC screening. *Intervention:* In the “optimized cancer screening” group, the intervention will combine sending invitation letters to non-adherent women with sending general practitioners (GPs) a list of their non-adherent patients. In the “standard cancer screening” group, the intervention will be limited to sending invitation letters to non-adherent women. In the “usual care” group, no letter will be sent either to women or to their GPs. *Primary endpoint:* CC screening test uptake will be assessed after a 6-month follow-up period. *Statistical analysis:* The percentage of women who are up-to-date with their screening at 6 months after the intervention will be compared across arms using a generalized mixed linear model.

**Discussion:**

A large-scale randomized trial of this nature is unprecedented. The study will enable us to assess a strategy relying on GPs, identified as the coordinators in this screening strategy. The study results should help policy makers to implement organized CC screening programs in the future.

**Ethics and dissemination:**

The study was approved was approved by the Ethics Committee of the National College of Teaching General practitioners (IRB00010804). It was recorded in ClinicalTrials.gov on the number NCT04689178 (28 December 2020). The study findings will be used for publication in peer-reviewed scientific journals and presentations in scientific meetings.

## Introduction

Cervical cancer (CC) ranks fourth among the most frequently diagnosed cancers among women worldwide (1). It is also the fourth leading cause of death from cancer in this population (2). In France, the CC incidence is close to 3,400 cases a year, and the mortality exceeds 1,400 deaths yearly (1). Incidence and mortality rates reach maximum values at age 45 and 50 respectively (3). CC remains one of the only cancers for which the prognosis is deteriorating in France (4). A slowing of the decrease in incidence has been observed since the 2000s (5). The five-year survival rate after diagnosis also decreased by 4 points (from 68 to 64%) between the periods 1998–1991 and 1991–2004 (4).

CC can be eradicated with vaccination and screening (2), and the World Health Organization (WHO) has defined a plan of action for the screening of 70% of women (6). Recommendations for CC screening are based on a cervical-uterine smear test to be carried out every 3 years. This screening can be performed during a visit to a general practitioner (GP), a gynecologist or a midwife. However, the rate of national screening coverage in France was only 58.2% over the 2017–2019 period, below the objectives defined by the WHO. ≪ The maximum screening rate was of 65.5% for the 35- to 39-year-olds, and progressively decreased to a minimum of 44.0% among 60- to 65-year-olds≫ (7).

To improve the quality of screening, health authorities in France have put forward the organization of a CC screening programme, based on three new innovations (8). The first consists of integrating a new tool into the screening strategy, the HPV test, which was not present in the previous recommendations. The second consists of sending a letter to women who have not undergone a smear test for over 3 years. The third targets reimbursement conditions by the French national health insurance system by making them more favorable (8).

The management of this programme is entrusted to regional organizations in charge of cancer screening. The regional organizations are in charge of collecting screening data for all women eligible for CC screening and follow-up in cases with a positive or abnormal result. These organizations have to send invitations and reminders by post to women aged 25 to 65 who have not spontaneously taken part in screening within the recommended intervals. The regional organizations collect information from health care providers (health insurance, pathology and cytology laboratories, hospital information systems, GPs and cancer registries) involved in screening.

In France, all patients over the age of 16 must follow a coordinated care pathway. The regular GP is at the center of this system. He must be consulted as a priority. The GP system is based on a reciprocal choice: that of the patient and that of the GP. The role of the GP in the initiation, implementation and follow-up of CC screening has been assessed (9). Several studies have tested different modalities of GP-based interventions to encourage CC screening (e.g. reminders in GPs' patient files, GP signed invitation/reminder letters, GP counseling (10–13), and provided inconclusive or heterogeneous results.

French GPs reported that having access to a list of their patients who are not up to date with their cancer screening (including CC screening) was one of the most valued attributes that would support their involvement in increasing their supply of cancer screening services. Supporting the rationale for this intervention, providing French GPs with a list of their patients who were not up-to-date with their colorectal cancer screening resulted in a small but significant 4.2% increase in patient participation in fecal immunochemical testing screening at 1 year (compared with patients receiving usual care) (14).

Thus in a context where organized screening restricts its communication to postal messages sent to women who have not had a smear test in the previous 3 years, we hypothesed that providing GPs with a list of their patients aged 40 or over who are not up-to-date in their screening could increase CC participation rate. Targeting these women is motivated by lower participation in CC screening (7) and higher incidence and mortality rates (3, 15).

The main research objective is to assess the effect of two modalities of organized CC screening programmes on CC screening uptake: (1) combining sending invitation letters to non-adherent women with sending GPs a list of their non-adherent patients (optimized cancer screening group), (2) sending invitation letters to non-adherent women only (standard cancer screening group), compared to (3) no intervention (usual care group).

The secondary objectives will be (1) to describe the types of tests carried out by health professionals, the pathological report results, and the healthcare pathways of women who undergo a screening test and (2) to analyse factors associated with lower screening test uptake.

## Methods and analysis

### Design and setting

The design is a cluster-randomized, open-label, controlled trial with 3 parallel groups, performed in the Loire-Atlantique geographical area (west coast of France). A cluster was defined as a group of GPs with the same primary practice address.

### Participants

All GPs practicing in the geographical area will be eligible. For the participating women, the inclusion criteria will be the following: aged 40 to 65 years at the beginning of the study, eligible for CC screening (no medical history of hysterectomy or CC), residing in Loire-Atlantique, and present on the patient list of a participating GP. The exclusion criteria for women will be either a medical history of hysterectomy or CC or having opted for a GP who declines participation in the study. The inclusion and exclusion criteria for women and GPs are shown in Table 1. The list of GPs and eligible women will be extracted from the regional organization in charge of cancer screening. The study design is based on the following estimations: 1,500 GPs and 100 000 women participants.

**Table 1:** Inclusion and exclusion criteria for GPs and women.

	**GPs**	**Women**
**Inclusion criteria**	Specialized in general medicine	40 to 65 years old
	Practicing in Loire-Atlantique	Residing in Loire-Atlantique
		Present on the patient list of a participating GP
		Affiliated with the health insurance system
**Exclusion criteria**	Refusal to take part in the study	Medical history of hysterectomy or CC
		Not present on the patient list of a participating GP

CC, Cervical Cancer; GP, General Practitioner.

### Randomization

At the beginning of the study, all GPs identified according inclusion and exclusion criteria were grouped into clusters according to their practice's address, to avoid contamination bias stemming from shared tracking mechanisms and communication among GPs within a practice. Then, a centralized randomization was performed on the clusters by the statistician in charge of the study, according to a 1:1:1 ratio, without stratification. The clusters, and consequently the GPs belonging to the clusters, and the patients affiliated with the GPs were then allocated to the 3 arms of the study.

### Intervention and control procedures

GPs will be randomly assigned to 1 of the 3 following groups.

In the “optimized cancer screening” group, the intervention will combine sending invitation letters to non-adherent women with sending GPs a list of their non-adherent patients. The invitation letters will be mailed by the regional organization in charge of cancer screening to non-adherent women, defined as women who have not undergone a Pap smear test in the last 3 years. The letter content will entail a recommendation to consult a health professional (a GP, a midwife, or a gynecologist) in order to receive a screening test. The letter will also state that the consultation and tests relating to CC screening will be free of charge (see Supplementary material S1). Postal letters mailed to GPs by the organization in charge of the cancer screening programme will provide one type of information: a nominative list of the given GP's female patients who have not received a PAP smear test in the last 3 years (from the list of women who are patients in his or her clinical practice) (see Supplementary material S4).

In the “standard cancer screening” group, the intervention will be limited to sending invitation letters to non-adherent women (8) (see Supplementary material S3).

In the “usual care” group, letters will be sent neither to women nor to their GPs, so the CC screening will be opportunistic.

Two successive waves of 30,000 letters to women (“optimized cancer screening” and “standard cancer screening” groups) were sent 1 month apart, to minimize the influx of requests for appointments (1,000 GPs).

### Objectives, primary endpoint, and judgement criterion

#### Main objective and primary endpoint

The main objective of this study is to assess whether sending an invitation letter to non-adherent women and a list of non-adherent women to GPs (“optimized cancer screening,” arm 1) can increase the proportion of women undergoing a screening test compared to the corresponding proportion observed after sending an invitation letter only to non-adherent women (“standard organized cancer screening,” arm 2) and that observed in the absence of any message (“usual care,” arm 3).

The primary endpoint is the proportion of women aged 40 to 65 who will be up-to-date with their screening (having performed a screening test in the last 3 years) 6 months after the intervention. The 6-month duration takes into account the capacities of professionals to respond to a consultation request for this type of medical procedure.

#### Secondary objectives and judgement criteria

##### Description of the types of tests carried out in first intention and the results of these tests in the 6 months after intervention

The first secondary objective was to describe the types of tests carried out and the test results 6 months after the intervention. In 2020, health authorities recommended an HPV test rather than a cervical-uterine smear test for women aged 30 to 65 (8). In this context, secondary judgement criteria will be the proportion of cytology examinations carried out among all tests performed, the proportion of HPV tests carried out among all tests performed, and the proportion of tests yielding abnormal results (cytology, HPV) among all tests performed.

##### Description of the types of tests performed to follow lesions detected by screening and the results of these tests in the 12 months following the intervention

The second secondary objective is to describe the types of tests carried out and the results from these tests in the 12 months after the intervention.

A “reflex” test is performed in second intention on the same initial screening sample following a positive or abnormal test; the result indicates the course of action to follow. In the case of a positive HPV test, the “reflex” test is cytological. In the case of abnormal cytology, the reflex test is one for HPV. The associated secondary judgement criterion is the percentage of “reflex tests” conducted among abnormal tests at 6 months.

The presence of lesions is checked for *via* colposcopy and confirmed by biopsy or cervical conization in cases with any abnormalities (8). A secondary judgement criterion will be the percentage of biopsies and conizations carried out among the abnormal screening tests in the 12 months following the intervention. The 12-month duration takes into account the time required for follow-up and for investigation procedures.

The most serious lesions are precancerous lesions (neoplasia, second and third-grade cervical intraepithelial neoplasia), *in situ* carcinomas and invasive cancer (16). A secondary judgement criterion will be the percentage of high-grade lesions detected (second, a third-grade cervical intraepithelial neoplasia, including *in situ* carcinomas and cancers) among the abnormal screening tests in the 12 months following the intervention.

##### Description of the treatments undergone by the women following abnormal screening tests in the 12 months after the intervention:

The third secondary objective is to describe the treatments undergone by women following abnormal screening tests in the 12 months after the intervention. The associated secondary judgement criterion will be the rate of treatments carried out (conization, laser, hysterectomy) among the abnormal screening tests in the 12 months following the intervention.

##### Description of the factors associated with lower participation in screening

The fourth secondary objective is to describe the factors associated with lower screening test uptake.

Many studies have reported the existence of social inequalities in screening (17). Some factors have been put forward: increased age (17), lower socio-economic status (18), multimorbidity (19, 20), irregularity of medical follow-up (21).

##### Description of the healthcare trajectory of women undergoing a screening test

The fifth secondary objective is to describe the healthcare trajectories of women undergoing a screening test.

An issue will be to determine which type of care provider will be consulted by women for a screening test. While the caregivers who perform screening tests are GPs, midwives or gynecologists, we will analyse the proportion of women who resort to a GP, a midwife or a gynecologist to undergo their screening test. The time required for women to resort to screening after having received the invitation to do so will also be determined. The time lapse will correspond to the time required to make an appointment and how easy it is to obtain an appointment with the professionals they contact. The judgement criterion will be the time period between posting the letter and the date of the screening test.

### Source of the data collected

The regional organization in charge of cancer screening will collect the data required to conduct the research from the following organizations: National Insurance System (patient administrative files, health professionals carrying out the medical procedures) and pathology and cytology laboratories (8). This collect will be carried out in accordance with the terms of reference defining the organization of organized CC screening in France (22). The transfer of data will be carried out in accordance with the data protection regulations that supervise personal data protection in the European Union.

The Supplementary material S4 summarizes the research objectives with the judgement criteria and provides details on the data collected and on the source of the data collected.

### Statistical analysis

All the data will be described globally and per randomization arm. The statistical unit will be the woman eligible for screening. The significance threshold will be set at 5%.

The analyses will be performed using R 3.6.0. software (R Foundation for Statistical Computing, Vienna, Austria. https://www.R-project.org/).

#### Main criterion

The percentage of women up-to-date with their screening at 6 months after the intervention will be compared across arms using a generalized mixed linear model with logit link function (logistic regression model that included a fixed effect for the arm and GP practice as a random effect), to adjust the analysis on the random effect of the GP *via* maximum likelihood, by adaptive Gauss-Hermite quadrature approximation (23).

A hierarchical procedure will be used to successively test the comparisons of the three arms, controlling type I error.

The first hypothesis tested will be the difference between the intervention arm (“optimized organized cancer screening”) and the “usual care” arm. If the test result is significant, a second test will be conducted, comparing the “optimized screening” arm with the “standard screening” arm.

#### Exploratory secondary analyses

The types of tests conducted and the types of professional consulted will be described and compared between arms using the same models as for the principal criterion. The time-lapse to screening uptake will be described and compared using a mixed Cox model adjusted for the type of professional chosen, the randomization arm and the GP as a random effect. The percentages of cytology tests and HPV tests conducted in the following 6 months and the percentage of abnormal tests among all the screening tests will be calculated and compared between the different arms using a generalized mixed linear model adjusted on the GP. The same will apply to the percentage of “reflex tests” conducted among the abnormal tests at 6 months, to the percentages of biopsies and conizations carried out among the abnormal tests in the ensuing 12 months, to the percentage of high-grade lesions detected (second- and third-grade cervical intraepithelial neoplasia, including *in situ* carcinomas and cancers) among the abnormal screening tests in the ensuing 12 months and to the percentages of women resorting to each type of professional.

A generalized mixed linear model will be used to assess factors associated with screening uptake among the following: age of the woman; economic status; chronic disease; Complementary health insurance status and French DEPrivation index of the place of residence (proxies of socio-economic status); and number of visits to GPs, midwives or gynecologists during the follow-up period.

#### Study population

The statistical analyses will be carried out for all randomized GPs and patients included in the study.

A sensitivity analysis will be conducted removing the following subjects from the analysis: patients for whom letters are returned (of the type “no longer at this address”), patients who have moved away from the Loire-Atlantique region, patients who have changed their GP, or patients whose GP has ceased working during the study period.

### Statistical justification of the sample size

Over the 2017–2019 period, the screening participation rate in Loire-Atlantique was 61.3% and concerned approximately 154 000 women (internal source: regional organization in charge of cancer screening). On the basis of data from the literature (10–12, 14), the research team estimates that the proportion of women who will undergo a screening test while not having had a smear test for over 3 years could reach 25%, thanks to the letter sent to their GP that includes the list of his or her non–up-to-date patients (“optimized” arm), whereas the increase in participation should be more limited in the “standard screening” arm (15%) and nil in the “usual care” arm. After intervention, participation rates should therefore be 71% in the “optimized screening” arm, 67.1% in the “standard screening” arm and 61.3% in the “usual care” arm.

In September 2020, there were 1,395 GPs in the Loire-Atlantique region, with an average of 2.15 GPs per medical practice. The multiplication factor derived from the cluster randomization is defined at 12.8 (intraclass correlation coefficient: 0.05). Inclusion should reach a minimum of 37,776 patients per arm to demonstrate a difference between the “optimized” arm and the “standard” arm. As a total of 154,000 eligible women will be included in the study, we will be able to demonstrate this difference with 51,300 women per arm.

### Status and study schedule

The start of the intervention will correspond to the dispatch of the letters to non-adherent women and their GPs (M0). The database concerning participation in organized screening in the following 6 months will be closed 24 months after the intervention (M24). The database concerning the types of tests carried out in the 12 months and their results will be closed 24 months after the intervention (M24). This schedule takes into consideration the time required for receipt of data and its coding by the regional organization in charge of cancer screening. All statistical analyses and valorisation of the results will be carried out from M24.

Table 2 presents the time schedule for enrolment, intervention and assessments.

**Table 2:** Time schedule for enrolment, intervention, and assessments.

	**Study period**									
	**Enrolment**	**Allocation**	**Post-allocation**							**Close out**
**Time point (month)**	**t _−1_**	**t_0_**	**t_1_**	**t_2_**	**t_3_**	**t_4_**	**t_5_**	**t_6_**	**…. t_24_**	
**Enrolment:**										
Eligibility for screening	**x**									
Informed consent	**x**									
Allocation		**x**								
**Interventions:**										
Optimized cancer screening			**x**	**x**	**x**	**x**	**x**		**x**	
Standard cancer screening			**x**	**x**	**x**	**x**	**x**		**x**	
Usual care			**x**	**x**	**x**	**x**	**x**		**x**	
**Evaluations:**										
Proportion of women aged 40 to 65 having undergone a screening test in the last 3 years at 6 months after the intervention.									**x**	
Proportion of cytological tests among all screening tests performed									**x**	
Proportion of HPV tests among all screening tests performed									**x**	
Proportion of abnormal test results (cytology, HPV) among all screening tests performed.									**x**	
Proportion of biopsies and conizations among abnormal screening test results at 12 months									**x**	
Proportion of high-grade lesions detected (second and third-grade cervical intraepithelial neoplasms including *in situ* carcinoma and cancers) among abnormal screening tests at 12 months									**x**	
Proportion of treatments performed (conization, laser, hysterectomy) among abnormal screening tests at 12 months									**x**	
Proportion of women having consulted a GP, a midwife or a gynecologist for a screening test									**x**	
Time lapse from dispatch of the invitation to the date of the screening test										**x**

### Data storage and conservation

All data concerning the included patients and GPs will be anonymised by the regional organization in charge of cancer screening before being sent to the research team. Data transfer methods will be those usually used *via* the specific secure channels. Data collected during the study will be kept in a computer file at Nantes University Hospital in compliance with the French personal privacy legislation dated 6 January 1978, amended by law n°2018-493 dated 20 June 2018, relating to the protection of personal data, and with the 2016/679 EU regulations and the 27 April 2016, ruling relating to personal data processing. Data processing will be carried out by Nantes University Hospital and registered in accordance with the regulations. Any information relating to the study will be kept for 15 years after the end of the study by Nantes University Hospital.

This study protocol has been prepared according to the 2013 SPIRIT guideline for clinical trial protocols (24) (see Supplementary material S5). It was recorded in ClinicalTrials.gov on the number NCT04689178 (December 28, 2020; Version 1 - September 14, 2020). The WHO Trial Registration Data Set Checklist is presented (see Supplementary material S6).

## Discussion

### Originality of the research

The proposed research is based on a randomized trial conducted on a large-scale population that is unprecedented in the field of CC screening.

Whereas only “prepost” studies have been carried out in French administrative areas (18, 25), this research should demonstrate an increase in screening uptake observed as a result of the implementation of a new approach to CC screening based on a letter sent to non–up-to-date women inviting them to visit a health professional to undergo a screening test. The study should also enable an assessment of whether sending a letter to the GP with a list of their patients aged 40 to 65 who have not undergone a cervical-uterine smear test for over 3 years could lead to an additional increase in screening uptake.

The international literature has helped with the design of this study. The meta-analysis by Cochrane de Staley et al. published in 2021 assessed the efficacy of interventions targeting women to promote screening participation. That study reported a significant uptake of CC screening following an invitation letter that was sent by post to women who were not up-to-date (9). Among the studies included in this meta-analysis, two randomized trials stood out for the number of inclusions. The first randomized trial included 58 780 women in the intervention arm and 29 919 in the control arm (26). It was carried out in 2002 in New Zealand, in an environment that is very different from the French context, since the proportion of smear test uptake was 4.44% at the end of the study in the intervention arm (women who had received a letter) vs. 2.90% in the control arm. The second randomized trial was conducted by Burack et al. in the USA in 2003 and included 2 471 women over 40 years of age (27). The trial explored the effect of combined reminders, by post and by messages in the medical records, concerning CC and breast cancer screening (compared to a reminder only for breast cancer screening). The women who were assigned to the combined intervention were more likely to undergo a smear test (30% in the intervention arm vs. 23% in the control arm). These studies are encouraging and present a strong argument for the implementation of a robust, large-scale investigation, such as the one we propose in this protocol.

Concerning the organization of care, the study will enable us to assess a strategy relying on GPs, identified as the coordinators in this screening strategy. It will also enable us to identify the healthcare trajectories of women who have been invited by a letter through the post to participate in CC screening.

This study will be performed in the Loire-Atlantique geographic area on the French west coast. It is opportune given the absence of any organized screening deployment in this area at the time this research protocol is being drafted. It is also particularly interesting because this area is already characterized by a higher participation in screening than the French national average (61.3% vs. 58.2% over the 2017-2019 period) (7).

### Expected results and benefits

Considering previous literature (10–12, 14), the increase in screening uptake could reach 25% and 15% in the optimized and standard cancer screening groups, respectively. The number of eligible patients identified (*n* = 94,393; 2020) should be sufficient to show a difference between the “optimized screening” and the “standard screening” arms. On the basis of an initial screening uptake of 61.3% in the general population in the third arm (“usual care”), increased participation could thus reach an absolute value of (1–0.61) × 25% in arm 1 and (1–0.61) × 15% in arm 2, corresponding to 9.7 and 5.8%, respectively.

In the context of this randomized study, the “usual care” arm will be monitored according to the usual practice of opportunistic screening. At the end of the 6-month period, the team plans to send invitations to the participants in arm 3, demonstrating the positive impact of organized screening.

### Robustness of the design and reproducibility of the intervention

The proposed research is characterized by the robustness of its design. Randomization will be carried out on GP medical practices with the aim of limiting confusion bias between arms. The study also stands out for its power; it is to be carried out on the scale of a whole French *department* (administrative unit). No randomized trial of this scope has been published in the literature exploring the impact of organized CC screening. Finally, the study is characterized by its reproducibility. This reproducibility will be documented, identifying the associated costs for the deployment of this intervention.

### Perspectives for health policies

Improving women's compliance with CC screening is a major issue that is necessary to decrease the incidence and mortality from cancer. The authors of this project believe that an increase in screening uptake will yield benefits in terms of morbidity and mortality in the population under study. The study results should help policy makers to implement organized CC screening programs in the future.

## Ethics and dissemination

### Ethics approval and consent to participate

The IMPACT-GP protocol (see Supplementary material S4) is in the field of Methodology Reference MR004, to which Nantes University Hospital conforms. On 27 November 2020, the protocol was approved by the Ethics Committee of the National College of Teaching General practitioners (IRB00010804) (see Supplementary materials S5, S6). Information about the deployment of the research will be given to GPs from the Loire-Atlantique by email from the regional organization in charge of cancer screening 6 months before the intervention. A letter of reminder to the GPs will inform all GPs randomized to the intervention arm that they can decline participation in the research by contacting the Data Protection Officer of the regional organization. The invitation letter will inform the patients that their personal data could be used for statistical purposes. The means to oppose inclusion are explained therein. The women who are not sent a letter will not be given any information about the way the study is to be conducted. Concerning this exemption, we refer to the international standards as defined by the IRB. ≪ (1) the research is designed to evaluate possible changes in public health programs, and (2) the research cannot practicably be carried out without the waiver. ≫ [Code of Federal Regulations. TITLE 45. Public welfare. Department of Health and Human Services. Part 46. Protection of human subjects, available at https://www.hhs.gov/ohrp/regulations-and-policy/regulations/45-cfr-46/index.html#46.116].

## Author contributions

CR and AG have full access to all the data in the study and take responsibility for the integrity of the data and the accuracy of the data analysis. Study concept and design: CR, A-SB, and AG. Acquisition, analysis, interpretation of the data, and statistical analysis: AG. Drafting of the manuscript: DT, LA, AG, and CR. Critical revision of the manuscript for important intellectual content: CG, CR, and A-SB. Administrative, technical, and material support: A-SB, CR, DT, and LA. Study supervision: CR. All authors contributed to the article and approved the submitted version.

## Funding

This article is supported by the French network of University Hospitals HUGO (Hôpitaux Universitaires du Grand Ouest).

## Conflict of interest

The authors declare that the research was conducted in the absence of any commercial or financial relationships that could be construed as a potential conflict of interest.

## Publisher's note

All claims expressed in this article are solely those of the authors and do not necessarily represent those of their affiliated organizations, or those of the publisher, the editors and the reviewers. Any product that may be evaluated in this article, or claim that may be made by its manufacturer, is not guaranteed or endorsed by the publisher.
