# Supplementary material for: Cervical cancer screening uptake: A randomized controlled trial assessing the effect of sending invitation letters to non-adherent women combined with sending their general practitioners a list of their non-adherent patients (study protocol)
Source: Front Public Health. 2022 Nov 10;10:1035288. doi: 10.3389/fpubh.2022.1035288 (PMC9686337; doi:10.3389/fpubh.2022.1035288)
Supplement: Supplementary file 1 [file Table_1.docx]

**Objectives, judgement criteria, data collection and source of the data collected**

| **Objectives** | | **Judgement criteria** | **Data collected** | **Source of the data collected** |
| --- | --- | --- | --- | --- |
| Main | To determine whether sending a letter of invitation to women not complying with screening and sending a list of noncompliant patients to their GPs (optimised screening group) **increases the CC screening uptake compared to that observed after**  . sending a letter of invitation to noncompliant women only (standard screening group),  . or sending no such invitation (usual care group). | . Proportion of women aged 40 to 65 having undergone a screening test in the last three years at 6 months after the intervention. | Dates of screening tests (if any) in the 36 months before inclusion and at 6 months after inclusion. | Regional organisation  in charge of cancer screening in Loire-Atlantique |
| Secondary | **To analyse the types of testing performed in first intention and the results of these tests at 6 months after the intervention** | . Proportion of cytological tests performed among all screening tests  . Proportion of HPV tests performed among all screening tests  . Proportion of abnormal test results (cytology, HPV) among all screening tests performed. | . screening test performed (cytology or HPV)  . abnormal test results (HPV + cytology) at 6 months. | Pathology and cytology laboratories |
|  | **To analyse the types of testing performed for follow-up of lesions detected and results of these tests at 12 months** | . Proportion of "reflex" tests performed (number of cervical smears after a positive HPV result) among abnormal test results at 6 months  . Proportion of biopsies and conizations performed among abnormal screening test results at 12 months.  . Proportion of high-grade lesions detected (second and third-grade cervical intraepithelial neoplasms), including in situ carcinoma and cancers) among abnormal test results at 12 months. | . reflex tests performed among abnormal tests at 6 months  . colposcopies performed at 12 months  . positive colposcopies  . biopsies performed  . conizations performed  . high-grade lesions detected (second and third-grade intraepithelial cervical neoplasms including in situ carcinoma and cancers) | Pathology and cytology laboratories |
| Secondary | **Description of treatment undergone by women following abnormal screening results, at 12 months after the intervention** | . Percentages of treatments performed (conization, laser, hysterectomy) among abnormal test results at 12 months. | Number of conizations, laser treatments and hysterectomies at 12 months. | Regional organisation  in charge of cancer screening in Loire-Atlantique |
| Secondary | **Description of factors associated with lesser compliance with screening** | . Participation rate according to age, socio-economic status, presence of chronic disease, and medical follow-up  . | Age of the woman, Proxies of socio-economic status: Complementary health insurance status and French DEPrivation index of the place of residence, Chronic diseases,, Number of visits to GPs, midwives or gynaecologists during the follow-up _period_ | National Insurance System Database |
|  | **Description of the care itineraries of women undergoing screening :**   - Description of the professionals resorted to by women for screening - Description of the time lapse to uptake of screening among noncompliant women following receipt of the invitation | The proportion of women having consulted a GP, a midwife or a gynaecologist for a screening test.  Time lapse between dispatch of the invitation and the date of the screening test. | Health professional carrying out the screening test (GP, midwife, gynaecologist).  Date of the screening test performed in the 6 months after inclusion. | National Insurance System Database |
