# Supplementary material for: Cervical cancer screening uptake: A randomized controlled trial assessing the effect of sending invitation letters to non-adherent women combined with sending their general practitioners a list of their non-adherent patients (study protocol)
Source: Front Public Health. 2022 Nov 10;10:1035288. doi: 10.3389/fpubh.2022.1035288 (PMC9686337; doi:10.3389/fpubh.2022.1035288)
Supplement: Supplementary file 2 [file Table_2.DOCX]

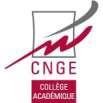
Comité Ethique du CNGE

155 rue de Charonne 75011 PARIS Courriel: [comite-ethique@cnge.Fr](mailto:comite-ethique@cnge.Fr)

Tél: 01 75 62 22 90

Paris, 27th November 2020,

Objective: Approval is sought from the CNGE Ethics Committee for the study: "GP IMPACT: Compliance with organised cervical cancer screening: A randomised study assessing the impact of letters addressed to GPs listing their patients who have not undergone a cervical smear examination in the last 3 years"

**AVIS 051120218**

**----------------------------------------------------------------------------------------------------------**

The Ethics Committee grants approval for the project: "GP IMPACT: Compliance with organised cervical cancer screening: A randomised study assessing the impact of a letter addressed to GPs listing their patients who have not undergone a cervical smear examination in the last 3 years"

**Cédric RAT for the Comité Ethique du CNGE**
