# Supplementary material for: Cervical cancer screening uptake: A randomized controlled trial assessing the effect of sending invitation letters to non-adherent women combined with sending their general practitioners a list of their non-adherent patients (study protocol)
Source: Front Public Health. 2022 Nov 10;10:1035288. doi: 10.3389/fpubh.2022.1035288 (PMC9686337; doi:10.3389/fpubh.2022.1035288)
Supplement: Supplementary file 3 [file Data_Sheet_1.DOCX]

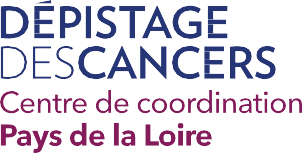


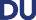

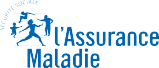

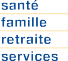

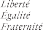

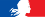

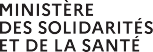


PDL_U


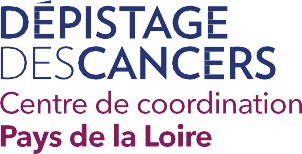


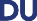


Case number:

Date of last smear:

Date of birth:

Social security number:

Caisse:


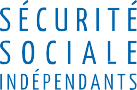

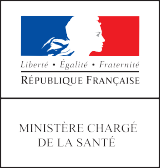

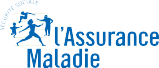

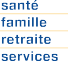


Dear Madam,

Screening for cervical cancer concerns women aged 25 to 65.

It enables early detection of any precancerous lesions and appropriate surveillance or treatment, and it guards against the later development of cervical cancer.

According to our information, it appears that you have not undergone this screening process for over 3 years. We would therefore like to invite you to make an appointment with a healthcare professional of your choice: a gynaecologist, a GP or a midwife.

The screening test is completely covered by health insurance with no outlay on your part. The consultation will be reimbursed by your health insurance organization in the usual manner.

Like any medical procedure, cervical screening entails benefits and limitations. We suggest that you read the leaflet enclosed carefully. If you require further information, you can talk with your gynaecologist, GP or midwife, and you can also discuss other preventive assessments or actions.

Your GP has been informed that you have received this letter.

We are of course available to answer your questions via the following number: **02 41 05 06 73.**

Dr Anne-Sophie Le Duc-Banaszuk

Physician coordinator

**If you are not eligible for this invitation, please return it duly completed with this statement on the reverse side. By responding, you will avoid receiving reminders that do not apply.**

**Invitation and labels to be presented to the practitioner at the time of the consultation. These will be used at the time of the screening test.**

**LABEL TO BE STUCK ON THE APPLICATION FORM**

**INVITATION NUMBER**

**For information relating to the processing of your personal data and your rights, see the reverse side of this letter.**

**I am ineligible for this invitation - I am completing the questionnaire**

*(on my own or with a medical practitioner)*

Name of my GP or midwife:...............................................................................................................

**I am ineligible for this invitation because:**

I was screened less than 3 years ago:

Smear test, on: [date]:

# J J M M A A A A

HPV test on: [date]:

# J J M M A A A A

I have no uterus (total hysterectomy not sparing the cervix [Date]):

# A A A A

And my GP, Dr............................................................................ confirmed that I did not need to be screened

Other reason (please detail):.....................................................................................................................................................

..............................................................................................................................................................................................

I do not wish to undergo this screening.*

Date:  **J J M M A A A A** Signature:

* I can alter my decision at any time by contacting the regional coordination centre for cancer screening at phone number 02 41 05 06 73.

1. **After completion, I am returning this document to Dr ………………….:**

Address………………………….

**Have you any questions?**

The Pays de la Loire CRCDC team will answer if you call the following number ….

**Information relating to the processing of your personal data and your rights.**

In relation to their mission of public interest, the French regional coordination centres for cancer screening (CRCDC) draft invitations and follow up persons concerned with the screening procedures. For this purpose, they collect data concerning your identity and your heath from the health insurance bodies and from relevant health professionals, and they store the data until the end of your follow-up. Should you oppose the processing of your data by the CRCDC, your data will be removed from the invitation file, and you will no longer be invited to undergo screening. You can exercise this right to oppose by writing to the Pays de Loire CRCDC at the following address: dpo@depistagecancers.fr. If you solely oppose the transmission of your examination results to the CRCDC, the centre will no longer take charge of the follow-up of your screening procedures, but you will remain in the invitation database, and you will continue to be invited to later screening campaigns. This so-called "partial" opposition should be reported to the health professional who carries out the screening test. In both instances, only the identification data will be transferred to the CRCDC to enable it to take into account your opposition. If you have not expressed opposition, the data required for your medical follow-up will be transferred to the health professional concerned. The data required for statistical assessments and calculations will be transferred to the public bodies in charge of these functions, among which Santé Publique France and the National Cancer Institute (INCa). These data will contribute to the INCa cancer database platform to enable the study of healthcare trajectories, to evaluate anticancer strategies and to conduct studies. There may be matching with the data in the national health database. For more information on the platform for cancer data, you can consult the INCa website: http://lesdonnees.e-cancer.fr/

In accordance with the French legislation on the protection of data and the legislation on computer processing and personal freedoms, you have a right to access, rectify, or remove your data and a right to the limitation of the processing of that data, rights that you can exercise by writing to the CRCDC in your region at the abovementioned address. You also have the right to file a request or complaint to the Commission National Informatique et Libertés if you consider that your rights are not respected.

🡄

Affix your identification label here
