# Supplementary material for: Cervical cancer screening uptake: A randomized controlled trial assessing the effect of sending invitation letters to non-adherent women combined with sending their general practitioners a list of their non-adherent patients (study protocol)
Source: Front Public Health. 2022 Nov 10;10:1035288. doi: 10.3389/fpubh.2022.1035288 (PMC9686337; doi:10.3389/fpubh.2022.1035288)
Supplement: Supplementary file 4 [file Data_Sheet_2.DOC]

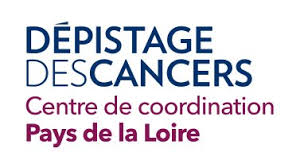


**Information about the study: IMPACT GP ***

*(*):* ***IM****proving* ***P****atient* ***A****dherence to* ***C****ervical cancer screening: A randomized* ***T****rial based on* ***GP*** *notification for women who have not undergone a PAP test in the last 3 years.*

Nantes, 15th January 2021

Dear colleague,

Screening for cervical lesions using the cervical smear technique has enabled a reduction in the incidence of cancer of the cervix and related mortality since the 1960s. Since 2010, this screening has been recommended for women aged 25 to 65.

Participation rates in this screening in the period 2016-2018 (*Public Health France -Santé Publique France*) in Loire Atlantique reached 60.7% despite an objective of over 80% in the 3^rd^ national cancer plan. In addition, a decrease of 10% has been observed for women after the age of 40.

The year 2020 was that of the deployment of organized screening in the Pays de Loire region. Such screening includes the follow-up of all pathological test results (cytology or HPV screening) and an alert in case of the absence of gynaecological follow-up. Alongside, a letter accompanied by labels for 100% insurance coverage for the test analysis is sent **solely** to women who have not undergone a smear test for over three years (approximately half of the women aged 25 to 65).

The *Regional Cancer Screening Coordination Centre* (*Centre Régional de Coordination des Dépistages des Cancers* (CRCDC)) -*Pays de la Loire*, the *Primary Health Insurance Fund of Loire-Atlantique (Caisse Primaire d’Assurance-Maladie de* *Loire-Atlantique)* and the *Department of General Practice* in the University of Nantes have designed the project IMPACT GP. This study aims to assess the usefulness of a complementary system that consists of sending GPs a list of the names of their patients aged 40 to 65 who have not undergone screening in the last 3 years.

This list is **practical assistance** to help you identify patients who have not been screened in the last three years.

The IMPACT GP study is a randomised study with three parallel arms aiming, after 6 months, to compare the impact of these two approaches for promoting participation in screening for cervical and uterine cancer among women aged 40 to 65: either 1) the dispatch of a simple targeted letter or 2) the dispatch of the same letter and a letter to the appropriate GPs accompanied by a list of their noncompliant patients.

Following randomisation, you have been assigned to the group of GPs who are to receive this **list of patients aged 40 to 65** who have not undergone a smear test for over 3 years. **The recommended screening test at this age is cervical-uterine sampling in liquid medium with screening for human papillomavirus (HPV) oncogenes.**

No extra workload is specifically asked of you. You are free to use the list made available to you as you deem appropriate, as follows:

• an alert on your professional software

• telephone reminders, or

• no particular action

The data collection will be conducted in the usual manner by the *Regional Cancer Screening Coordination Center* (CRCDC) *Pays de Loire.*

We would like you to note any inaccuracies in the enclosed list and would be grateful for your help in improving it by indicating the patients who are up-to-date in their screening or are otherwise ineligible: for instance, because of a past total hysterectomy or because the patient has not been seen for a long time.

The extraction of the data to compile this list dates from November 2020. The tests run thereafter were not taken into account. The data are derived from reimbursements by the health insurance systems (CCAM codes registered), which has an impact on your ROSP data (*remuneration based on public health objectives* – payments for public health objectives)

You are free to refuse the analysis of your data via a letter to the following address: dpo@depistagecancers.fr

We are available for any further information you might require.

For the Steering Committee:

Dr Anne-Sophie Le Duc-Banaszuk

Regional coordinating doctor

CRCDC-Pays de la Loire

Pr Cédric Rat

Department of General Practice

University of Nantes

INSERM U1232 – team 2
