# Supplementary material for: Cervical cancer screening uptake: A randomized controlled trial assessing the effect of sending invitation letters to non-adherent women combined with sending their general practitioners a list of their non-adherent patients (study protocol)
Source: Front Public Health. 2022 Nov 10;10:1035288. doi: 10.3389/fpubh.2022.1035288 (PMC9686337; doi:10.3389/fpubh.2022.1035288)
Supplement: Supplementary file 7 [file Data_Sheet_5.DOCX]

World Health Organization Trial Registration Data Set

| DATA CATEGORY | INFORMATION |
| --- | --- |
| **Primary Registry and Trial Identifying Number** | ClinicalTrials.gov NCT04689178 |
| **Date of Registration in Primary Registry** | December 28, 2020 |
| **Secondary Identifying Numbers** | Nantes CHU: RC20-0364 |
| **Source(s) of Monetary or Material Support** | Not applicable |
| **Primary Sponsor** | Nantes CHU |
| **Secondary Sponsor(s)** | Not applicable |
| **Contact for Public Queries** | Delphine Teigné, University Hospital of Nantes, Research Department, Nantes, France |
| **Contact for Scientific Queries** | Cédric Rat, National Institute for Health and Medical Research/INSERM U1302 Team 2, CRCINA, Nantes, France |
| **Public Title** | The efficacy of interventions towards women aiming to promote cervical screening |
| **Scientific Title** | Cervical cancer screening uptake: A randomised controlled trial assessing the effect of sending invitation letters to nonadherent women combined with sending their GPs a list of their nonadherent patients. (Study Protocol). IMPACT-GP |
| **Countries of Recruitment** | France |
| **Health Condition(s) or Problem(s) Studied** | Cervical cancer |
| **Intervention(s)** | **"optimised cancer screening" group** : the intervention will combine sending invitation letters to nonadherent women with sending GPs a list of their nonadherent patients.  "**standard cancer screening" group** : the intervention will be limited to sending invitation letters to nonadherent women  **"usual care" group** : letters will be sent neither to women nor to their GPs, so the CC screening will be opportunistic |
| **Key Inclusion and Exclusion Criteria** | **Key inclusion**  GPs:   - Specialized in general medicine, - Practising in Loire-Atlantique   Women:   - 40 to 65 years old, - Residing in Loire-Atlantique, - Present on the patient list of a participating GP, - Affiliated with the health insurance system   **Exclusion criteria**  GPs:   - Refusal to take part in the study   Women:   - Medical history of hysterectomy or CC - Not present on the patient list of a participating GP |
| **Study Type** | The study is a cluster-randomized, open-label, controlled trial with 3 parallel groups with 3 parallel groups, designed to evaluate the effect of two modalities of organised CC screening programmes on CC screening uptake: 1) sending invitation letters to nonadherent women 2) sending GPs a list of their nonadherent patients (optimised cancer screening group |
| **Date of First Enrollment** | 8th of january 2021 |
| **Sample Size** | 154 000 eligible women |
| **Recruitment Status** | Complete |
| **Primary Outcome(s)** | Outcome Name: Screening  Method of measurement: Proportion of women aged 40 to 65 who having performed a screening test in the last 3 years  Timepoint : 6 months after the intervention. |
| **Key Secondary Outcomes** | Outcome Name: types of tests and results of these tests  Method of measurement : proportion of cytology examinations carried out among all tests performed, the proportion of HPV tests carried out among all tests performed, and the proportion of tests yielding abnormal results (cytology, HPV) among all tests performed  Timepoint : 6 months after the intervention.  Outcome Name: types of tests performed to follow lesions detected by screening and the results of these tests  Method of measurement : percentage of “reflex tests” conducted among abnormal tests at 6 months, percentage of biopsies and conizations carried out among the abnormal screening tests, percentage of high-grade lesions detected (second, a third-grade cervical intraepithelial neoplasia, including in situ carcinomas and cancers) among the abnormal screening tests  Timepoint : 12 months following the intervention.  Outcome Name: factors associated with lesser compliance with screening  Method of measurement : Participation rate according to age (over 50), income (women with low incomes qualifying for basic health coverage) and comorbidities.  Timepoint : 6 months after the intervention.  Outcome Name: healthcare trajectory of women undergoing a screening test  Method of measurement : proportion of women having consulted a GP, a midwife or a gynaecologist for a screening test, Time lapse between dispatch of the invitation and the date of the screening test  Timepoint : 6 months after the intervention. |
| **Ethics Review** | Protocol approved by the Ethics Committee of the National College of Teaching General practitioners (IRB00010804). |
| **Completion date** | Data collection not completed |
| **Summary Results** | Data analysis not started |
| **IPD sharing statement** | Plan to share IPD : No |
