# Supplementary material for: Cervical cancer screening uptake: A randomized controlled trial assessing the effect of sending invitation letters to non-adherent women combined with sending their general practitioners a list of their non-adherent patients (study protocol)
Source: Front Public Health. 2022 Nov 10;10:1035288. doi: 10.3389/fpubh.2022.1035288 (PMC9686337; doi:10.3389/fpubh.2022.1035288)
Supplement: Supplementary file 8 [file Data_Sheet_6.DOC]

**Protocol IMPACT GP**

IMPACT GP: ***IM****proving* ***P****atient* ***A****dherence to* ***C****ervical cancer screening:* **Cervical cancer screening uptake: A randomised controlled trial assessing the effect of sending invitation letters to non-adherent women combined with sending their GPs a list of their non-adherent patients.**

RESEARCH

RESEARCH PROTOCOL NOT INVOLVING PEOPLE IN VIEW OF DEVELOPING BIOLOGICAL AND MEDICAL KNOWLEDGE

**Coordinating investigator:**

**Pr. Cédric RAT**

Département de Médecine Générale, Université de Nantes

INSERM U1232 – équipe 2

1 rue Gaston Veil – 44000 NANTES

Fax 02.40.82.67.76

[cedric.rat@univ-nantes.fr](mailto:cedric.rat@univ-nantes.fr)

**Second coordinating investigator:**

**Dr Anne Sophie BANASZUK**

Centre Régional de Coordination de Dépistage des Cancers

5 Rue des Basses Fouassières, 49000 Angers

Fax 02.41.24.02.85

as.banaszuk@depistagecancers.fr

**Methodologist:**

**Mme Aurélie Gaultier**

Direction de la Recherche - Département Promotion

Hôpital St Jacques - CHU de Nantes

5, allée de l'île Gloriette- 44093 Nantes cedex 1

aurelie.gaultier@chu-nantes.fr

[aurelie.gaultier@chu-nantes.fr](mailto:aurelie.gaultier@chu-nantes.fr)

**Promoter:**


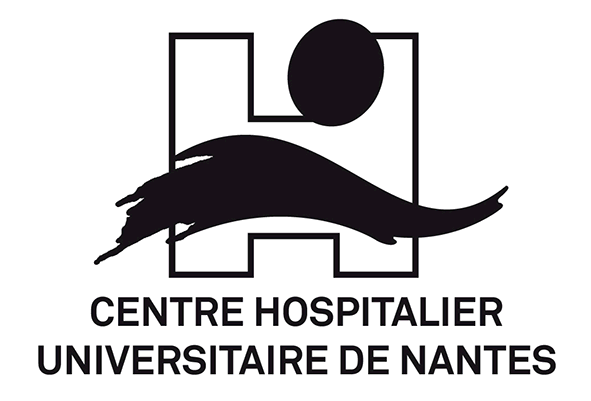
**CHU de Nantes**


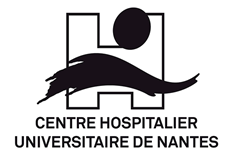
Direction des Affaires Médicales

et de la Recherche
5, allée de l’île Gloriette
44 093 Nantes cedex 01 (FRANCE)

Fax: 02 53 48 28 36

| Project context |
| --- |

| **1/ Background:**  ***Incidence, mortality, advantages of early screening for cervical lesions***  The development of screening tests for cervical lesions using a smear test has enabled a decrease in cervical cancer incidence and mortality since the 60s(1). Incidence has been estimated at 2 920 new cases per year (1). However, certain indicators are not satisfactory. The number of women failing to undergo smear tests remains high. Certain factors are associated with not undergoing a smear test (2): age over 50, precarious socio-economic status, comorbidities (particularly diabetes, depression, cancer, hepatitis and/or HIV), disability, dependence and/or being accommodated in an institution. Because of late diagnoses, prognosis has deteriorated among women over 50 years of age: the 5-year survival rate has decreased from 68% in 1991 to 64% in 2004 (3). Mortality remains high among women with lower socio-economic status.(3)  ***A national decision: implemention of organised screening tests for cervical cancer***  A recent systematic review of the literature reaffirmed the impact on mortality of organised screening for cervical cancer (4). In France, the year 2020 saw a generalised implementation of organised screening at national level.  The Plan Cancer 2014-2019 (3) brought about several evolutions:  1) The French regional centres for the coordination of cancer screening (centres régionaux de coordination de dépistage des cancers (CRCDC)) are now required to collect data relating to screening tests for cervical cancer undergone by women between 25 and 65 years of age. The French national health insurance is to provide reimbursement data so that smear tests can be identified as having been carried out, and histocytopathology centres are to provide the results of these tests. Follow-up of pathological smear tests and HPV positive tests concerns all women, whether they have received the targeted information letter or not (see 2).  2) The regional centres for the coordination of cancer screening are to ensure that letters are sent to women who have not undergone a smear test in the last three years.  3) The screening test of reference among 30-65 year-old women is replaced by the HPV test, carried out every 5 years (whereas screening was based until now on smear tests every 3 years). (5)  4) Several evolutions related to reimbursement have been planned in the setting of this organised screening process. Presentation of the letter sent by the CRCDC will enable a 100% cover for screening tests. Cover for HPV tests as part of organised screening has been in force since the summer 2020 following an amendment of the 2018 specifications. (6)  ***Organised screening should enable an increase in participation from 51% to 62%*** (7)  Organised screening has been the subject of experiments in several French pilot regions (8). A program of organised screening was launched in 1994 in the Bas-Rhin *département,* and extended to the whole of the Alsace region in 2001. Year after year, the proportion of women having undergone a screening test in the previous 3 years has increased, reaching 70%, against 57% on a national scale. Analysis over a longer period shows that the proportion of women having had a smear test in the last 5 years reached 83% (9). This experiment was continued in the Isère, Doubs and Martinique *départements*. In 2010, the experiment was extended to 9 other *départements* (13.4% of the French population). An analysis over the 2010-2012 period showed that the proportion of women having had a smear test in the last 3 years was 60.8% in these *départements*: 51.4% of the women had not received a letter from the screening management centre because their smear test had been performed less than 3 years previously (8). Among the women who had been invited, 17.5% had a screening test in the 12 months following the invitation and 12.1% had it in the twelve months following the reminder. While organised screening enabled an increase in the proportion of women undergoing a smear test, the observed participation rates did not reach the objective of 80% set by the third Plan Cancer implementers (3).  ***The Loire-Atlantique département: specificities at the time of the implementation of organised screening***  The deployment of organised screening in the Loire-Altantique *département* was planned for the last quarter of 2020 for women aged 30-65.  In the Loire-Atlantique, for the 2015-2017 period, the proportion of women between 25 and 65 having had a smear test in the last three years was 59.2%. Analysis of the data provided by Santé Publique France showed that participation was high up to 40 years of age (66%) but it decreased by more than 10 points, falling to 55.8%, for the age ranges after 40. (10)  The Plan Cancer 2014-2019 encouraged the reinforcement of the GPs’ role in initiation, implementation and follow-up of screening. In Loire-Atlantiqe, the medical density of GPs enabled patients to attend consultations: localised potential accessibility (an indicator showing the possibility of access to a practitioner) for private practice GPs aged under 65 was 4 medical appointments per inhabitant per year, which is slightly more than the national average of 3.7 consultations (11).  **2/ Theoretical concepts, previous experiences on which the project is based**  ***Le role of GPs in the screening process***  Among the leads to increase participation, Sicsic *et al.* reported in a 2019 publication that GPs wished to have at their disposal a list of their non-adherent patients who were not up-to-date in their screening (12). Organised screening, as it is intended, solely provides for communication by post with the patients. Implementing a specific organisation that enables GPs to have a list of their patients who have not had a smear test in the last 3 years could be relevant. Because of their particular position with their patients, their relational skills, and their skills in tackling the obstacles and levers enabling changes in behaviour, GPs could play a central role in increasing participation. In the project we plan to carry out, we believe that a substantial increase in participation could be attained by combining the advantages of a systematic approach (coordinated by the CRCDC) with the advantages of a more individualised approach, centred on patients supported by their GP.  ***Studies pointing to the usefulness of involving GPs***  The hypothesis of the favourable effect of an intervention consisting in providing GPs with a list of their patients who have not had a smear test in the last 3 years has been supported by a few studies  In a randomized study by Somkin *et al.* in 1997 in the USA (3564 women included), organised screening increased participation by about 10% and invitations via GPs enabled a 3% gain in further participation (13).  Another randomized study carried out in Italy in 1998 (2 028 women included) showed that invitations to women from their GP were associated with a higher participation rate, compared to invitations from the screening centre (OR =1.13 [1.05,1.21]) (14).  Finally, a previous randomized trial carried out in Nantes (conjointly with Nantes University Hospital, Nantes General Medicine Centre, the regional centre for organised screening and Social Security) showed that providing GPs with a list of patients not up-to-date with their colorectal cancer screening increased participation in FIT screening tests, compared to invitations via the regional coordinating centre for cancer screening. (15)  ***The need for a randomized trial in general population, to experiment on the provision of a list of the names of non-adherent women to their GP***  We believe that this type of intervention could be experimented in France to reinforce the organisation of screening tests among women aged between 40 and 65 who have not had a smear test for more than 3 years.  Even in the French *départements* where organised screening has been implemented, participation remains much lower than the objective of 80%, fixed at national scale, so that any strategy aiming to reinforce screening tests appears as an important step to assess.  Although there are pilot studies, they are only very few: we only found 3 studies that assessed the impact of resorting to GPs to increase cervical cancer screening. These studies are not recent: they date back more than 20 years. Of the 3 studies, one only included 85 women (so that it concluded to the absence of a significant impact, but probably due to a lack of power (OR = 1.69[0.75,3.82])(16). These studies were carried out in countries where the health systems are different from the French system. The study by Somkin *et al*. (13) showed a favourable effect on participation, but comparisons appeared difficult since participation in screening tests was found to be very low in the control arm: 9.1%. (The most comparable study could be the study by Segan *et al.* (14), in Italy, where participation in the control arm was evaluated at 41%.)  ***A randomized trial showing the impact on participation, unprecedented in France***  In this project, we also hope to provide greater insight into the advantages of organised screening.  The meta-analysis by Jansen et al(4) in 2020 showed that there has never been a randomized trial in Europe providing information on the impact of organised screening for cervical cancer on mortality. The meta-analysis by Cochrane de Everett *et al.* (2011) analysed randomised clinical trials assessing the impact of organised screening on participation. (17). Twelve trials (99 651 women included) explored organised screening using an invitation letter of the type planned in France (Invitation letter vs. control). The meta-analysis concluded to a significant increase in participation as a result of letters being sent to non-adherent women: OR = 1.44 [1.24-1.67]. However, the randomized trial with the largest number of women (Morrell *et al.* 2005: 58 780 women in the intervention arm and 29 919 in the control arm) was conducted in New-Zealand (18), a very different environment from the French context, as smear test rates reached 4.44% at the end of the study in the intervention arm (vs. 2.90% in the control arm). The second largest randomized trial was conducted by Burack *et al.* in the USA in 2003 and included 2 471 women over 40 years of age (19). The results showed an increase in participation in screening by smear test, with a proportion of adherent women reaching 30% in the intervention arm (vs. 23% in the control arm).  Whilst the French context is characterized by a much higher initial participation rate than that found in the international literature (20) (45,4% average cover in Europe, with great discrepancies between countries, from 9.2% in Romania to over 80% in certain Scandinavian countries), one of the objectives is to assess increase in participation, as promoted by organised screening tests in the Loire-Atlantique *département*, where participation (59.2%) was higher than the national average over the 2015-2017 period (58,7%) (10).  In general, while some studies focusing on the “before – after” effect have been conducted in other French *départements* in pilot experiments, the originality of the present study is the organisation of a randomised trial in France, and the opportunity afforded by the fact that the organised screening is not yet functioning in the Loire-Atlantique département.  **3/ Developing actions in the light of previous experiences**  **and 4/ Partnerships**  Nantes University general medicine department  The staff from the Nantes University general medicine department will guarantee the scientific design of the project, will ensure the scientific coordination, and will be in charge of the scientific valorisation. The project will be integrated into the main research axis of the general medicine department “Prevention and Screening in Oncology”, where research is conducted in close collaboration with the INSERM UMR 1232 team 2. As Pr. Cédric Prat, the IMACT GP project coordinator, heads the general medicine department in Nantes University and is a member of the Nantes/Angers oncology research centre, he is in a good position to ensure coordination among participants in Team 1, formed for the project.  The regional centre for the coordination of cancer screening in the Pays de la Loire  The proposed study will draw on the quality of collaboration between the above-mentioned research teams and the regional centres for the coordination of cancer screening in the Pays de la Loire. Within the framework of previous projects, close relationships have been created between Nantes University and teams from the CRCDC. This excellent collaboration has enabled many research projects to be achieved in our territory.  The CRCDC has several missions in the deployment of organised screening for cervical cancer:   - To characterise the population aged 25 to 65 eligible for screening, to define exclusions, and to manage the corresponding files, - To collect an exhaustive list of screening test and histology results (smear tests, HPV tests, follow-up examinations) for all women eligible for screening in the Pays de la Loire, - To manage letters sent to patients not up-to-date with their screening, - To organise communication and awareness programmes for different health professionals.   Dr Anne Sophie BANASZUK, the regional coordinator of the regional centre for cancer screening, will also co-coordinate the IMPACT GP project. She will ensure optimal coordination between the participants, under the heading Team 2, formed for the project.  The University Hospital (CHU) and the support to research in primary care by Nantes CHU and the clinical research directorate  The IMPACT GP project will have the benefit of support generated by the need for structuring in primary care research in the Loire-Atlantique-Vendée region. Nantes University and the CHU have proposed an organisation to ensure promotion of primary care research, and this organisation received a launch budget from 2017 to 2020, jointly provided by the French regional health agency and the Pays de la Loire Conseil Régional. The structure implemented today constitutes a genuinely original force in the Nantes area. The CHU provides a project manager 100% dedicated to primary care projects, led conjointly with the GPs. Experience has shown that this project manager is an asset in the development of projects, guaranteeing implementation and follow-up. He provides expertise from the CHU clinical research management and the necessary support for regulatory and budget monitoring and contracting. The structure has also enabled a post of methodologist/statistician in primary care to be set up.  In this partnership focusing on the issues of structuring in primary care research, the CHU will provide its experience and logistics.  French national health insurance  Every 3 months, the French health insurance bodies issue an update of files concerning women aged 25 to 65 in the Loire-Atlantique and in the region. In the file concerning the organised screening for cervical cancer population, the name of the attending GP chosen by the patient is indicated. Health insurance bodies also issue a file with all reimbursements for smear tests, HPV tests, biopsies, conizations and total hysterectomies every 3 months.  Previous experiences of successful collaborations  The IMPACT GP project will draw on the different collaborators’ working experiences acquired in the course of different projects previously successfully conducted in collaboration, resulting in numerous publications. Two projects were interventional studies in primary care in the field of cancer screening.  . The COPARIME study on melanoma screening, included about 4 000 patients with high-risk for melanoma, recruited in primary care, and it enabled a description of their follow-up after 5 years of evolution. It was funded by Inca. It yielded 7 international publications (21) (22) (23) (24) (25) (26) (27) (28).  The IDLN study was something of a model for the present project. It was an interventional randomized study in general population, and the design included several aspects in common with the project we are promoting for cervical cancer screening. This study included more than 30 000 patients and more than 1 400 practitioners. It was funded by Inca. The results yielded 2 international papers published in JAMA (15) (29). The first results concerned patient participation in colorectal cancer screening (original article published in 2017). A new publication was accepted in April 2020 (again in JAMA) giving an account of the favourable impact of screening on cancer incidence. The publication was planned for the June 16^th^ issue.  **In all**, the existence of these direct, operational links among different participants in the project (research team members, general practitioners, methodologists-statisticians, practitioners from the regional centre for the coordination of organised screening, advisory physcians and personnel from the health insurance bodies) is an asset for the IMPACT GP project.  On organisational level, a work consortium on research development in primary care is being finalised between Nantes University, Nantes CHU and the French national health insurance. (The CRCDC is liable to adhere to this consortium in the coming months). The aim is to encourage the development of shared expertise in the field of primary care, with a view to achieving common projects.    The partnerships between these teams will enable numerous skills to be combined:   - Scientific expertise, defining of issues, expertise on the themes of cervical cancer screening (Nantes University general medicine, CRCDC PDL, cancer registry in the Loire-Atlantique-Vendée region, midwifery school in Nantes CHU) - Expertise on database handling and implemention of organised screening (CRCDC PDL, health insurance), - Methodological expertise in conducting clinical trials on primary care populations in the field of cancer screening, regulatory know-how, clinical researchers and statisticians - Expertise on scientific valorisation (INSERM U1232, Département de Médecine Générale de Nantes)   Pr. Cédric PRAT, coordinator of the project, will be the link across all these entities. He will ensure the best possible coordination among these teams.  **5/ Role of the project in this context (particularly in terms of strategy, coordination, innovation, and reproducibility).**  The originality of the randomized trial planned in this project is that it sets out to ascertain whether the fact of sending GPs a list of their patients who have not undergone a smear tests in the last 3 years will enable an increase in participation in organised screening in the Loire-Atlantique département, where the participation rate is already higher than the national average.  In scientific terms, the present study will be characterized by:  . The robustness of the design, based on a randomized trial on an unprecedented scale in the field of cervical cancer screening in France,  . Its power, since the study will be conducted on the scale of the whole population of a large *département*.  . Its reproducibility, as the design is in intention-to-screen, and as the intervention can be easily reproduced.  Regarding the organisation of care, the present study will enable the assessment of a strategy implicating GPs identified as the coordinators in the screening process. It will enable an evaluation of the health trajectories of the women who have been invited by letter to take part in cervical cancer screening.  In terms of research dynamics, this project will enable the reinforcement of combined work dynamics among the main players in care strategies and screening in primary care: GPs, the CRCDC, health insurance bodies and Nantes CHU.  **Bibliography**  1. Defossez G, Le Guyader‑Peyrou S, Uhry Z, et al. Estimations nationales de l’incidence et de la mortalité par cancer en France métropolitaine entre 1990 et 2018. Synthèse. Saint‑Maurice: Santé publique France, 2019. 20 p.  2. Barré S, Massetti M, Leleu H, et al. Caractérisation des femmes ne réalisant pas de dépistage du cancer du col de l’utérus par frottis cervico-utérin en France. Bull Epidémiol Hebd. 2017;2-3:39-47. http://invs.santepubliquefrance.fr/beh/2017/2-3/2017_2-3_3.html.  3. Plan cancer 2014-2019. Guérir et prévenir les cancers: donnons les mêmes chances à tous, partout en France. Paris: Ministère des Affaires sociales et de la Santé, Ministère de l’Enseignement supérieur et de la Recherche; 2014. 150 p. http://www.e-cancer.fr/Plan-cancer/Plan-cancer-2014-2019priorites-et-objectifs.  4. Jansen EEL, Zielonke N, Gini A, et al. Effect of organised cervical cancer screening on cervical cancer mortality in Europe: a systematic review. Eur J Cancer. 2020;127:207‑23.  5. Évaluation de la recherche des papillomavirus humains (HPV) en dépistage primaire des lésions précancéreuses et cancéreuses du col de l’utérus et de la place du double immuno-marquage p16/Ki67 [Internet]. Haute Autorité de Santé. [cité 18 mai 2020]. Disponible sur: https://www.has-sante.fr/jcms/c_2806160/fr/evaluation-de-la-recherche-des-papillomavirus-humains-hpv-en-depistage-primaire-des-lesions-precancereuses-et-cancereuses-du-col-de-l-uterus-et-de-la-place-du-double-immuno-marquage-p16/ki67  6. République Française. Décision du 4 mars 2020 de l'Union nationale des caisses d'assurance maladie relative à la liste des actes et prestations pris en charge par l'assurance maladie | Disponible sur: https://www.legifrance.gouv.fr/jo_pdf.do?id=JORFTEXT000041751664  7. Bourdillon F, Ifrah N. Éditorial. Dépistage du cancer du col de l’utérus: des évaluations pour mieux l’organiser. Bull Epidémiol Hebd. 2017;(2-3):24-5. http://invs.santepubliquefrance.fr/ beh/2017/2-3/2017_2-3_0.html.  8. Duport N, Beltzer N. Dépistage organisé du cancer du col de l’utérus évaluation du dépistage organisé du cancer du col de l’utérus dans les quatre départements pérennes et les neuf départements expérimentaux. Expérimentation 2010-2014. Santé Publique France. 2016.  9. Baldauf JJ, Fenderc M, Bergerond C. Cervical morbidity in Alsace, France: results from a regional organized cervical cancer screening program. European Journal of Cancer Prevention. 2019;28:33-39.  10. Etat des lieux de Santé Publique France des taux de participation aux frottis 2015-2017 d’après les Données SNIIRAM avant le déploiement national du dépistage organisé.  11. ORS Pays de la Loire. Démographie des médecins en Pays de la Loire : généralistes. Situation 2018 et évolution. La santé observée. 2019;8.4 p.  12. Sicsic J, Franc C. Préférences des médecins généralistes vis-à-vis des mesures incitatives associées aux dépistages des cancers. Santé Publique 2019; s2(HS2):33-41. doi:10.3917/spub.197.0033.  13. Somkin CP, Hiatt R, Hurley L, et al. The effect of patient and provider reminders on mammography and papanicolaou smear screening in a large health maintenance organization. Arch Intern Med. 1997;157:1658.  14. Segnan N, Senore C, Giordano L, et al. Promoting participation in a population screening program for breast and cervical cancer: a randomized trial of different invitation strategies. Tumori 1998;84:348-53.  15. Rat C, Pogu C, Le Donné D, et al. Effect of physician notification regarding nonadherence to colorectal cancer screening on patient participation in fecal immunochemical test cancer screening: a randomized clinical trial. JAMA 2017;318:816-24.  16. Bowman J, Sanson-Fisher R, Boyle C, et al. A randomised controlled trial of strategies to prompt attendance for a Pap smear. Journal of Medical Screening 1995;2:211-8.  17. Everett T, Bryant A, Griffin MF, et al. Interventions targeted at women to encourage the uptake of cervical screening. Cochrane Database Syst Rev 2011;5. Disponible sur: https://www.cochranelibrary.com/cdsr/doi/10.1002/14651858.CD002834.pub2/full/fr  18. Morrell S, Taylor R, Zeckendorf S, et al. How much does a reminder letter increase cervical screening among under-screened women in NSW? Aust N Z J Public Health. 2005;29:78‑84.  19. Burack RC, Gimotty PA, Simon M, et al. The effect of adding Pap smear information to a mammography reminder system in an HMO: results of randomized controlled trial. Prev Med. 2003;36:547‑54.  20. Sauvaget C, Weiderpass E. Éditorial. Éradication du cancer du col utérin : une priorité de santé publique. Bull Epidémiol Hebd. 2019;22-23:408-9. http://beh.santepubliquefrance.fr/ beh/2019/22-23/2019_22-23_0.html.  21. Rat C, Quereux G, Rivière C, et al. Impact of a targeted screening on melanoma prevention behaviour. A randomised controlled trial. Annals of Family Medicine 2014;1:21-8.  22. Rat C, Quereux G, Monegier du Sorbier M,et al. Patients at elevated risk of melanoma: individual predictors of non-compliance to GP referral for a dermatologist consultation. Preventive Medicine 2014;64:48-63.  23. Rat C, Quereux G, Grimault C, et al. Melanoma incidence and patient compliance in a targeted melanoma screening intervention. One-year follow-up in a large French cohort of high-risk patients. European Journal of General Practice. 2015;21:124-30.  24. Rat C, Grimault C, Quereux G, et al. Proposal for an annual skin examination by a general practitioner for patients at high risk for melanoma: a French cohort study. BMJ Open 2015;5:e007471.  25. Rat C, Quereux G, Grimault C, et al. Inclusion of populations at elevated risk of advanced melanoma in a pilot targeted screening project involving general practitioners. Scandinavian Journal of Primary Healthcare 2016;34:286-94.  26. Rat C, Houd S, Quereux G, et al. Skin cancer prevention and screening: what is the role played by general practitioners? BMJ Open 2017;7:e013033.  27. Rat C, Dreno B, Nguyen JM. Why we should focus on melanoma targeted screening strategies. Dermatology. 2017;233:480-481.  28. Rat C, Hild S, Gaultier A, et al. Control locus and socio-demographic factors associated with participation in a pilot targeted screening program for melanoma: a survey. BMJ Open 2017;7:e016071.  29. Schmeltz H, Rat C, Pogu C, et al. Effect of physician notification regarding nonadherence to colorectal cancer screening on cancer early detection: A randomized clinical trial. JAMA. In Press.  30. Hild S, Johanet M, Valenza A, et al. Quality of decision aids developed for women at average risk of breast cancer eligible for mammographic screening: systematic review and assessment according to the International Patient Decision Aid Standards instrument. Cancer 2020. doi: 10.1002/cncr.32858. |
| --- |

| Project objectives and finalities |
| --- |
| **1/ Detailed objectives, with figures whenever possible**  **Principal objective:**  The main research objective is to assess the effect of two modalities of organised CC screening programmes on CC screening uptake: 1) combining sending invitation letters to non-adherent women with sending GPs a list of their non-adherent patients (optimised cancer screening group), 2) sending invitation letters to non-adherent women only (standard cancer screening group), compared to 3) no intervention (usual care group).  In view of the previous literature (13,14,15,16), the increase in screening uptake could reach 25% and 15% in the optimised and standard cancer screening groups respectively. The number of eligible patients identified (n= 94 393; 2020) should be sufficient to show a difference between the “optimised screening” and the “standard screening” arms. On the basis of an initial screening uptake of 61.3% in the general population in the third arm ("usual care"), increased participation could thus reach an absolute value of (1–0.61) × 25% in arm 1 and (1–0.61) × 15% in arm 2, corresponding to 9.7% and 5.8% respectively. Calculation of the NNT for the present study is included in the statistics section*. 2. Statistical justification of the number of inclusions.*  **Secondary objectives:**  *Description of the types of tests carried out in first intention and the results of these tests in the 6 months after intervention.*  The first secondary objective is to describe the types of tests carried out and the test results 6 months after the intervention. In 2020, the health authorities recommended an HPV test rather than a cervical-uterine smear test for women aged 30 to 65. In this context, the secondary judgement criteria will be the proportion of cytology examinations carried out among all tests performed, the proportion of HPV tests carried out among all tests performed, and the proportion of tests yielding abnormal results (cytology, HPV) among all tests performed.  *Description of the types of tests performed to lesions detected by screening and the results of these tests in the 12 months following the intervention.*  The second secondary objective is to describe the types of test carried out and the results from these tests in the 12 months after the intervention.  A “reflex” test is performed in second intention on the same initial screening sample following a positive or abnormal test; the result indicates the course of action to follow. In the case of a positive HPV test, the “reflex” test is cytological. In the case of abnormal cytology, the reflex test is for HPV. The associated secondary judgement criterion is the percentage of “reflex tests” conducted among the abnormal tests at 6 months.  The presence of lesions is checked for via colposcopy and confirmed by biopsy or cervical conization in cases with any abnormalities. A secondary judgement criterion will be the percentage of biopsies and conizations carried out among the abnormal screening tests in the 12 months following the intervention. The 12-month duration takes the time required for follow-up and for investigation procedures into account.  The most serious lesions are pre-cancerous lesions (neoplasia, second and third-grade cervical intraepithelial neoplasia), in-situ carcinomas and invasive cancers. A secondary judgement criterion will be the percentage of high-grade lesions detected (second, a third-grade cervical intraepithelial neoplasia, including in-situ carcinomas and cancers) among the abnormal screening tests in the 12 months following the intervention.  *Description of the treatments undergone by the women following abnormal screening tests in the 12 months after the intervention*  The third secondary objective is to describe the treatments undergone by women following abnormal screening tests in the 12 months after the intervention. The associated secondary judgement criterion will be the proportions of treatments carried out (conization, laser, hysterectomy) among the abnormal screening tests in the 12 months following the intervention.  *Description of the factors associated with lower participation in screening*  The fourth secondary objective is to describe the factors associated with lower screening test uptake.  Many studies have reported the existence of social inequalities in screening (2). Certain factors have been put forward: educational level, income, socio-professional category, comorbidities and age (2).  *Description of the healthcare trajectory of women undergoing a screening test.*  The fifth secondary objective is to describe the healthcare trajectories of women undergoing a screening test.  One issue will be to determine which type of care provider women will contact for a screening test. The caregivers who perform screening tests can be GPs, midwives or gynaecologists, and we will therefore analyse the proportion of women who resort to a GP, a midwife or a gynaecologist to undergo their screening test. The time required for women to resort to screening after having received the invitation to do so will also be determined. This time lapse will correspond to the time required to make an appointment and how easy it is to obtain an appointment with the professionals they contact. The judgement criterion will be the time between posting the letter and the date of the screening test.  **2/ Qualitative aspects targeted**  /  **3/ Expected benefits**  The first expected benefit is an increase in participation rates in screening tests by about 20% in the arm with a list sent to GPs and by about 15% in the arm with only the letter to the patient.  The increase in participation will enable the detection of pathological lesions to be improved: 5% for pathological smear tests and 10% for oncogenic HPV tests. The expected gynaecological monitoring is over 90% with follow-up or adequate treatment of high-grade lesions,  Our study is in complete coherence with the 2019-2024 Cancer Plan, and the authors of this project believe that an increase in screening participation will yield benefits in terms of morbidity and mortality in the target population.  **4/ Future perspectives for the project: shared action, larger-scale deployment or deployment on national level, publications planned ….**  The deployment of organised screening over the whole national territory in 2020 should enable an increase in the proportion of women having undergone a screening test in the last 3 years. Our study sets out to complement this screening system by providing GPs with a practical guidance tool. As non-adherence is multi-factorial, the fact of giving GPs a list of non-adherent women should help increase the efficacy of the letters sent to patients in the organised screening framework.  The present study is based on a simple and fairly inexpensive design, so that a generalisation of the suggested intervention will be feasible if the effects prove positive.    The present study combines the benefits of a systematic approach coordinated by an impersonal structure (the CRDC) and the benefits of an approach centred on patients and supported by their GP. Our study proposes an intervention that capitalises on the special position occupied by GPs towards their patients. Our intervention reinstates their position in the screening process, in coherence with the expectations expressed in previous research. |

| Project implementation |
| --- |

| **METHODOLOGY**  **General aspects**  This will be an open, prospective, randomised controlled trial with three parallel arms.  The study will concern all GPs in the Loire-Atlantique *département*, France applying the inclusion and non-inclusion criteria, and their female patients aged from 40 to 65 years who are eligible for cervical cancer screening.  The randomisation unit will be the medical practice, identified by its address. Randomisation will thus be performed on the place of practice rather than the practitioner to avoid confusion bias between arms. Thus several practitioners practising in a given place of practice will be assigned to the same arm, either "List to GP + letter to patients", "letter to patients alone", or "screening on individual initiative ".  The statistical unit will be the patient.  The analysis will be intention-to-treat. The study will be conducted in "real life" conditions taking account (and reporting on) of the fact that a given intervention has variable impact depending on the practitioners targeted by the intervention.  **Study population**  Inclusion criteria  The GPs eligible for this study will be individuals who do not have *Mode d'Exercice Particulier* status (complementary and alternative medicine), practising in Loire Atlantique. The list of the GPs in this territory will be derived from CRCDC Pays de Loire (cancer screening coordination centre). The population of eligible GPs is estimated to amount to 1500.  Eligible patients will have the following characteristics:  - Age 40 to 65  - Affiliated to a health insurance system  - Present on the list of a participating GP  - Residing in Loire Atlantique.  In practice, the list of patients will be derived from CRCDC Pays de Loire; their database collects information from the different health insurance bodies (*Régime Général, Mutualité Sociale Agricole*, other).  In Loire Atlantique the target population was estimated in 2019 to amount to 233 500 women (French public health data).  Non-inclusion and exclusion criteria  The GPs will receive information on the implementation of the survey 3 months before it starts, and will be able to contact the CRCDC data protection delegate if they wish to refuse to participate. The GPs who refuse to participate will not be included and thus will not be assigned to any arm in the process of randomisation (see also section on ethics and non-opposition).  Patients with a history of total hysterectomy and those with a pathological smear result will not be eligible for screening and excluded.  Women who have not named an attendant GP (*médecin traitant*) will not be included. In Loire-Atlantique the percentage of insured individuals who have not named their attendant GP has been estimated to be between 7 and 15% on the basis of health insurance data, the figure being variable from one territory to another. The percentage specifically aggregating the population of women aged 40 to 65 years is not available).  **Randomisation, intervention arms and control arm**  Randomisation will concern medical practices, so that practitioners will be distributed across the three arms.  On the basis of the list of practitioners assigned to the different arms, the CRCDC will draw up a list of names of women eligible for cervical screening aged 40 to 65 for each GP, excluding patients meeting non-inclusion criteria.  Thus when randomisation is complete, participating GPs and their patients will be randomly assigned to one of the three following arms:  "optimised cancer screening" group  • The GPs will receive a list of their patients aged 40 to 65 who have not undergone cervical cancer screening in the last three years, with specific attention to factors of non-participation.  • The patients will receive a letter: 1) inviting them to obtain an appointment with a health professional (GP, midwife, gynaecologist) to undergo a screening test, and 2) specifying that the screening procedure cost will be covered 100%.  "standard cancer screening" group  • The GPs in this arm will not receive the list of their patients aged 40-65 who have not undergone cervical cancer screening in the last three years.  • The patients will receive a letter: 1) inviting them to obtain an appointment with a health professional (GP, midwife, gynaecologist) to undergo a screening test, and 2) specifying that the screening procedure cost will be covered 100%.  "usual care" group  • The GPs in this arm will not receive the list of their patients aged 40-65 who have not undergone cervical cancer screening in the last three years.  • The patients will not receive a letter from CRCDC. The consultation and the test will be reimbursed as normal by the health insurance system and the complementary insurance.  **NB** The patients aged 40-65 who have not undergone cervical cancer screening in the last three years will be identified by way of the absence of health insurance reimbursement for the intervention.  In practice, the list of patients will be derived from the Pays de Loire CRCDC database, which draws its information from the health insurance bodies (*Régime Général, Mutualité Sociale Agricole*, other).  For Loire-Atlantique, the number of patients aged 40 to 65 who have not undergone a cervical smear test in the last three years was estimated in 2019 to be 130 300 (public health data).  **Main and secondary judgement criteria**  A. **The primary endpoint** is the proportion of women aged 40 to 65 who are up-to-date with their screening (having performed a screening test in the last 3 years) 6 months after the intervention. The 6-month duration takes into account the capacities of professionals to respond to a consultation request for this type of medical procedure.  **B. Secondary judgement criterion**  - the proportion of cytology examinations carried out among all tests performed, the proportion of HPV tests carried out among all tests performed, and the proportion of tests yielding abnormal results (cytology, HPV) among all tests performed.  - the percentage of “reflex tests” conducted among abnormal tests at 6 months.  - the percentage of biopsies and conizations carried out among the abnormal screening tests in the 12 months following the intervention.  - the percentage of high-grade lesions detected (second, a third-grade cervical intraepithelial neoplasia, including in situ carcinomas and cancers) among the abnormal screening tests in the 12 months following the intervention.  - the rate of treatments carried out (conization, laser, hysterectomy) among the abnormal screening tests in the 12 months following the intervention.  - Participation rate according to age (over 50), income (women with low incomes qualifying for basic health coverage) and comorbidities.  - the proportion of women having consulted a GP, a midwife or a gynaecologist for a screening test.  - the time period between posting the letter and the date of the screening test.  **Data collection**  The data will be collected or extracted via complementary, coordinated means:  **• Some of the data will be collected from CRCDC** via routine methods:  - Identification of patients eligible for organised screening who have named an attending GP meeting the inclusion criteria for GPs  - Patients having undergone screening or an HPV test (date of the examination in this case) in the 12 months following inclusion.  - Patient's address and IRIS code so as to attribute a European Deprivation Index score.  The study will process data transferred between the health insurance body and CRCDC as defined in the national references: "Types and contents of files required for the creation of databases for cervical cancer screening" (INCa September 2018).  Administrative and socio-demographic data enabling the characterisation of the women:  • Family name • Given name(s) • Date of birth (DD/MM/YYYY) • Address • Post code, town • Health insurance body • Email • Named attending GP • Identification number of the named practitioner • Universal health cover status (CMU) (yes/no).  Variables enabling identification, for each woman, of the medical acts performed in the setting of cervical cancer screening:  • Date of the act • Code of the act • Identification number of the practitioner performing the act • Identification number of the prescribing practitioner   - Data specifically collected from the health insurance system:   . Characteristics of the GPs: age, gender, classification of the practice locality, activity in numbers of acts per year, convention sector (1,2), practising alone or in a group  . Patient characteristics: CMU status, long-term illness (ALD) and CS status (assistance in contracting a complementary health insurance) (with a view to adjusting analyses of these variables).  . Number of consultations with a GP/ gynaecologist a) in the 24 months before the dispatch of the invitation letter and b) in the 12 months following the dispatch of the letter.  **Statistical analysis**  **1. Description of the statistical methods planned**  All the data will be described globally and per randomisation arm. The statistical unit will be the woman eligible for screening. The significance threshold will be set at 5%.  The analyses will be performed using R 3.6.0. software (R Foundation for Statistical Computing, Vienna, Austria. URL <https://www.R-project.org/>.)  **Main criterion**  The percentage of women up-to-date with their screening 6 months after the intervention will be compared across arms using a generalised mixed linear model to adjust the analysis on the random effect of the GP.  A hierarchical procedure will be used to successively test the comparisons of the three arms, controlling for type I error.  The first hypothesis tested will be the difference between the intervention arms (“optimised organised cancer screening” and “standard organised cancer screening”) and the "usual care" arm. If the result is significant, a second test will be conducted, comparing the "optimised screening" arm with the "standard screening" arm.  **Exploratory secondary analyses**  The types of tests conducted and the types of professional consulted will be described and compared between arms using the same models as for the main criterion. The time-lapse to screening uptake will be described and compared using a mixed Cox model adjusted for the type of professional chosen, the randomisation arm and the GP as a random effect. The percentages of cytology tests and HPV tests conducted in the following 6 months and the percentage of abnormal tests among all the screening tests will be calculated and compared between the different arms using a generalised mixed linear model adjusted on the GP. The same will apply to the percentage of "reflex tests" conducted among the abnormal tests at 6 months, to the percentages of biopsies and conizations carried out among the abnormal tests in the ensuing 12 months, to the percentage of high-grade lesions detected (second- and third-grade cervical intraepithelial neoplasia, including in situ carcinomas and cancers) among the abnormal screening tests in the ensuing 12 months and to the percentages of women resorting to each type of professional.  A generalised mixed linear model will be used to assess factors associated with screening uptake among the following: age of the woman; low economic status; long-term illness status; the French DEPrivation index of the place of residence; and the number of visits to GPs, midwives or gynaecologists during the follow-up period.  **2. Statistical justification of the sample size**  Over the 2017-2019 period, the screening participation rate in Loire-Atlantique was 61.3% and concerned approximately 154 000 women (internal source: regional organisation in charge of cancer screening). On the basis of data from the literature, the research team estimates that the proportion of women who will undergo a screening test while not having had a smear test for over 3 years could reach 25%, thanks to the letter sent to their GP that includes the list of his or her non–up-to-date patients ("optimised" arm), whereas the increase in participation should be more limited in the "standard screening" arm (15%) and nil in the "usual care" arm. After the intervention, participation rates should therefore be 71% in the "optimised screening" arm, 67.1% in the "standard screening" arm and 61.3% in the "usual care" arm.  In September 2020, there were 1 395 GPs in the Loire-Atlantique, with an average of 2.15 GPs per medical practice. The multiplication factor derived from the cluster randomisation is defined at 12.8 (intra-class correlation coefficient: 0.05). Inclusion should reach a minimum of 37 776 patients per arm to demonstrate a difference between the "optimised" arm and the "standard" arm. As a total of 154 000 eligible women will be included in the study, we will be able to demonstrate this difference with 51 300 women per arm.  **3. Randomisation**  The practices of the GPs will be randomised in the three parallel arms (cluster randomisation) with a 1:1:1 ratio. Each GP will be linked to the randomisation arm and his or her place of practice, and each patient to the randomisation arm of her attending GP.  **Piloting of the project and the roles of the different players**  **Steering Committee**  The project will be coordinated by Pr Cédric RAT.  Dr Anne Sophie Banaszuk is the project deputy coordinator.  The collective expertise is ensured by the Steering Committee which will gather all the partners  This Committee will define the general organisation and deployment of the project, will coordinate information and will decide on how to proceed in case of unexpected events.   - Cédric RAT, project coordinator for Nantes University - Anne Sophie BANASZUK, deputy project director, for CRCDC Pays de la Loire - Emmanuelle BATAILLE, in charge of statistical studies, for the health insurance body   **Roles of the different people involved**  Department of general medicine  Throughout the study, the coordination will be ensured by Cédric RAT (Nantes University, INSERM U 1232, team 2). He will be in charge of the design and drafting of the final protocol for the randomised trial, the drafting of intermediate reports and the final scientific report, and of the scientific valorisation of the trial.  CRCDC  CRCDC represented by Dr Anne-Sophie Banaszuk will ensure the study coordination.  It will take part in all stages in the research, from the design of the protocol for the randomised trial to its final scientific valorisation. It will be in charge of collecting information from the different sources, and of dispatch of the letters to the eligible women and the GPs. It will have the task of updating the status of each patient with respect to organised cervical cancer screening. The CRCDC will be in charge of the database. It will be in charge of the transfer of data to the research teams.  The Nantes CHU (university hospital) clinical research directorate (DRC)  The Nantes CHU DRC methodologist and biostatistician will ensure that the methodology of the statistical analyses is complied with. She will be in charge of drawing up the randomisation list, of the analysis of all the data from the randomised trial using the anonymised data transferred by CRCDC.  The DRC teams will be in charge of legal procedures and requirements.  Health Insurance bodies  As in the IDLN study ([15,29] a partnership will be established with the health insurance bodies.  They will provide the CRCDC with the data that is routinely transferred, and will also include certain data specifically collected in the setting of the present study (see Data Collection section)  The health insurance bodies will provide their expertise in the identification of medical acts and the tracing of healthcare trajectories using information in their databases.  **Legal requirements, ethics committee and non-opposition by participants**  The Jardé legislation does not apply to this study. The research does not involve human beings directly, since it aims to evaluate modes of practice among health professionals.  1. Computerised data and CNIL approval  The data processing will be registered in the Nantes CHU data protection body (RGPD).  The data collected in the course of the study will be stored in a computerised file in compliance with the "informatique et libertés" legislation of January 6^th^ 1978, modified by legislation n° 201#8-493 dated 20^th^ June 2018 relating o the protection of personal data, and with the European parliament legislation 2016/679 and that of the European Council dated 27^th^ April 2016 relating to the protection of individuals with respect to the processing of personal data and the free circulation of that data.  The protocol belongs to the field of methodology (reference MROO4 with which Nantes CHU complies.)  2. Ethics approval  The experimental protocol will be evaluated in view of the ethical requirements. The study does not come under Jardé[[1]](#footnote-2) requirements. The project will be submitted to prior approval by the Ethics Committee assessing non-RIPH projects like that of the Collège National des Généralistes Enseignants (CNGE).  3. The principle of non-opposition in the setting of a study aiming top assess new procedures in public health.[[2]](#footnote-3)  At least 3 months before the first inclusion, the GPs will be informed by letter about this pilot study on cervical cancer screening. They will be invited to signal their refusal if they do not wish to participate. In the absence of explicit refusal by a GP, he or she will be included in the study.  This mode of collection of non-opposition will be conducted in compliance with international ethical requirements for studies aiming to assess new procedures in the area of public health. International recommendations stipulate that explicit consent can be waived when the study concerned meets the following: 1) The research is designed to evaluate possible changes in public health programmes, and 2) The research cannot practicably be carried out without the waiver.  The mode of implementation will follow that agreed by CNIL, CPP and various international players (clinicaltrials.gov, JAMA) for the IDLN study previously funded by the National Cancer Institute (France)  **Data collection and anonymity**  CRCDC- Pays de la Loire will collect the study data.  Initially, the CRCDC Pays de Loire computing department (ZEUS database) will be in charge of the request to form the populations for the three arms.  The data will be centralised by CRDCDC.  The methods of transfer of data between the health insurance and the regional cancer screening coordination centre will be those in routine use.  CRCDC will transfer an anomymised database comprising a patient identifier code, but no data identifying the person, to the person in charge of the analysis (dates replaced by periods)  **Data collection - quality control**  CRCDC will collect data over the 12 months following the intervention.  CRCDC- Pays de la Loire will produce the data described above, drawing on the data transferred by the health insurance.  **Management and analysis of the data and valorisation**  The data analysis will be conducted under the responsibility of the study methodologist and biostatistician, on a database transferred by CRCDC. The valorisation of the results will be conducted via collaboration among the individuals involved in the study, commensurately with their implication and responsibilities at the different stages.  **Project implementation calendar**  **Provisional calendar**  The project is planned to run over 36 months.  **M-4** **to M-2:**  - Legal procedures, registration of the study in Nantes CHU RGPD register, submission of the project to the ethics committee, registration of the trial on clinicaltrials.gov.  - The health insurance body transfers to CRCDC the list of non-MEP GPs practising in Loire Atlantique (this information circuit is already established between the health insurance bodies and CRCDC).  - Transfer to CRCDC by the different health insurance bodies of information relating to women aged 40 to 65 residing in Loire Atlantique (this information circuit is already established between the health insurance bodies and CRCDC):  . Name, first name, date of birth, Social Security number, address of place of residence (data that is routinely transferred), name of GP  . Presence of a medical act enabling identification of a cervical cancer screening test (according to standard codes) in the course f the last 3 years  . ALD (long illness), CMU (universal health cover) and ACS (complementary health cover allowance) status.  **M-2 à M-0**:  - CRCDC generates an inclusion code per medical practice and transfers the coded list to the methodologist and statistician.  -The methodologist statistician carries out the centralised randomisation of the addresses of places of practice of GPs to 3 parallel arms, and transfers the result of the randomisation to CRCDC. This procedure avoids the transfer of names attached to data to the coordinator's team. The name-linked data remains with CRCDC, as is usual with respect to CNIL requirements.  **M0-M2**: Intervention in arms 1 and 2:  In the arm "lists to GPs AND letter to patients"  . For patients not having undergone cervical smear in the last 3 years dispatch of the letter inviting them to undergo a screening test covered 100% by the health insurance  AND  . Parallel dispatch to each GP of a list of the names of their patients who have not undergone a smear test in the last 3 years  In the arm "Letter to patients":  . For patients not having undergone cervical smear in the last 3 years dispatch of the letter inviting them to undergo a screening test covered 100% by the health insurance  No list of patients is however sent to the GPs.  In the arm "Individual screening initiative ":  . No letter is sent to women who have not undergone a screening test in the last 3 years  . No patient lists are sent to the GPs.  **M10**: Closure of the database relating to follow-up data for the main criterion (occurrence of a screening test)  **M10 to M12: Analysis of the main judgement criterion – intermediate report on participation**  **M20**: Closure of the database relating to anatomo-pathological data and other secondary criteria. Transfer by CRCDC to the coordinator of a file with anonymous individual data relating to participating patients.  **M20 to M26**: **Analysis of the secondary criteria** concerning anatomo-pathological data, the care trajectory, and factors associated with lesser participation.  **M26 to M29:** Drafting of the final report  **M29 to M32:** Drafting of scientific articles and follow-up to publication   | **Actions** | **M-4 to M-2** | **M-2 to M-0** | **M0 to M2** | **M10** | **M10 to M12** | **M20** | **M20 to M26** | **M26 to M29** | **M29 to M32** | | --- | --- | --- | --- | --- | --- | --- | --- | --- | --- | | Legal procedures | X |  |  |  |  |  |  |  |  | | Identification of eligible women by CRCDC | X |  |  |  |  |  |  |  |  | | **Randomisation** of GPs to 3 parallel arms |  | X |  |  |  |  |  |  |  | | **Intervention**: dispatch of letters in arms 1 and 2 |  |  | X |  |  |  |  |  |  | | Closure of database for main criterion |  |  |  | X |  |  |  |  |  | | **Analysis of main criterion** |  |  |  |  | X |  |  |  |  | | Closure of database for secondary criteria |  |  |  |  |  | X |  |  |  | | **Analysis of secondary criteria** |  |  |  |  |  |  | X |  |  | | Drafting final report |  |  |  |  |  |  |  | X |  | | Scientific valorisation |  |  |  |  |  |  |  |  | X | |
| --- | --- | --- | --- | --- | --- | --- | --- | --- | --- | --- | --- | --- | --- | --- | --- | --- | --- | --- | --- | --- | --- | --- | --- | --- | --- | --- | --- | --- | --- | --- | --- | --- | --- | --- | --- | --- | --- | --- | --- | --- | --- | --- | --- | --- | --- | --- | --- | --- | --- | --- | --- | --- | --- | --- | --- | --- | --- | --- | --- | --- | --- | --- | --- | --- | --- | --- | --- | --- | --- | --- | --- | --- | --- | --- | --- | --- | --- | --- | --- | --- | --- | --- | --- | --- | --- | --- | --- | --- | --- | --- | --- | --- | --- | --- | --- | --- | --- | --- | --- | --- | --- | --- | --- | --- | --- | --- | --- | --- | --- | --- |
| Evaluation of results |
| **1/ Expected results**  Our study should lead to an increase in uptake of organised cervical cancer screening as a result of sending GPs a list of their patients aged 40 to 65 who have not undergone a test in the last 3 years in comparison to 1) only sending a letter to women who are not up to date (routine in organised screening) or 2) no letter sent to GPs or their patients.  In view of the previous literature (13,14,15,16), the increase in screening uptake could reach respectively 25% and 15% in the optimised and standard organised cancer screening groups. The number of eligible patients identified (n= 94 393; 2020) should be sufficient to show a difference between the “optimised screening” and the “standard screening” arms. On the basis of an initial screening uptake of 61.3% in the general population in the third arm ("usual care"), increased participation could thus reach an absolute value of (1–0.61) × 25% in arm 1 and (1–0.61) × 15% in arm 2, corresponding to 9.7% and 5.8%, respectively. The calculation of the NNT (number needed to treat) we propose to use is set out in the section Statistics – justification of the numbers to include.  At a time when organised screening is being set up on a national scale:   - This project will provide a tool to back up practice among GPs, in the context of the decision in the national Cancer Plan to make GPs the coordinators of screening. - The project adds to the various modes of intervention available to decision-makers to increase uptake of cervical cancer screening. - This project enables the evaluation of the trajectories of women who undergo a test after receiving the letter while they have not undergone one for the last 3 years. It will enable a description of resort to care in terms of the professionals consulted and the time-lapse to access to care. - This project finds its place in the dynamic of organised screening on the scale of a regional territory (Loire Atlantique), where the participation rate is already higher than the national rate. - This project affords a unique opportunity to analyse the effects of the implementation of organised screening using a scientific method - A robust design, based on a randomised trial, unprecedented in the area of cervical cancer screening. - A study with considerable power, since the analysis concerns the whole population of a whole *département*. We found no other study on this scale in the literature. - Our project will produce results that will be reproducible, since the design is in intention to screen, and the intervention tested is straightforward and inexpensive - With respect to the research dynamic, this project will back up the dynamics of collaborative work among the main players in care strategies and screening in primary care: GPs, CRCDC, the health insurance bodies, the CHU   **2/ Method of evaluation**  The method of evaluation used will be a reference scientific method deploying a large-scale randomised trial for the main criterion. The other evaluation criteria (secondary) will be the subject of scientific analyses according to reference methods (see also the section Statistic).    The participation rate will be compared across arms using generalised linear models to take account of the cluster randomisation.  The proportion of abnormal smear results and positive HPV tests, and the time to consultation according to the professional chosen, will be described and compared across the three arms.  Factors associated with non-participation will be analysed using multivariate linear regression.   - Evaluation of the participation rates at 6 months:   Participation rate = number of screening tests (cervical smear or HPV test) / Number of patients targeted by organised screening   - Results of screening tests - Total number of cervical smears or HPV tests with results - % of unsatisfactory cervical smear tests /HPV tests - % of positive cervical smear tests /HPV tests - The screening activity per speciality will be described, reporting the number of cervical smears performed and the number of HPV tests per practitioner speciality: GP, gynaecologist, midwife, other (laboratory)     **3/ Indicators for project follow-up and for results, mode of evaluation of the quality of the action undertaken, etc**  Project follow-up indictors:  Compliance with the different stages in implementation as described in the project calendar  Results indicators:  Number of women eligible for screening  Number of letters sent to arms 1 and 2  Number of screening tests performed at 6 months  Number of abnormal test results in the 3 arms.  Evaluation of the quality of the actions undertaken  The study we propose is a large-scale randomised trial in the general population, with a robust design and very good reproducibility*.* |

1. Jouannin A, Mamzer MF, De Fallois M, et al. Validation d’un outil de classement de la recherche à destination des internes de médecine générale d’après la loi « Jardé ». Exercer, la revue francophone de médecine générale 2019 [↑](#footnote-ref-2)
2. Code of Federal Regulations. TITLE 45. PUBLIC WELFARE. DEPARTMENT OF HEALTH AND HUMAN SERVICES. PART 46. PROTECTION OF HUMAN SUBJECTS, available at https://www.hhs.gov/ohrp/regulations-and-policy/regulations/45-cfr-46/index.html#46.116 [↑](#footnote-ref-3)
